# The Role of Instrumental Variables in Causal Inference Based on Independence of Cause and Mechanism

**DOI:** 10.3390/e23080928

**Published:** 2021-07-21

**Authors:** Nataliya Sokolovska, Pierre-Henri Wuillemin

**Affiliations:** 1 NutriOmics, UMR S 1269, INSERM, Sorbonne University, 91, Boulevard de l’Hôpital, 75013 Paris, France; 2 Laboratoire d’Informatique de Paris 6, Sorbonne University, 4 Place Jussieu, 75005 Paris, France; pierre-henri.wuillemin@lip6.fr

**Keywords:** common hidden cause, graphical models, probabilistic models

## Abstract

Causal inference methods based on conditional independence construct Markov equivalent graphs and cannot be applied to bivariate cases. The approaches based on independence of cause and mechanism state, on the contrary, that causal discovery can be inferred for two observations. In our contribution, we pose a challenge to reconcile these two research directions. We study the role of latent variables such as latent instrumental variables and hidden common causes in the causal graphical structures. We show that methods based on the independence of cause and mechanism indirectly contain traces of the existence of the hidden instrumental variables. We derive a novel algorithm to infer causal relationships between two variables, and we validate the proposed method on simulated data and on a benchmark of cause-effect pairs. We illustrate by our experiments that the proposed approach is simple and extremely competitive in terms of empirical accuracy compared to the state-of-the-art methods.

## 1. Introduction

Causal inference purely from non-temporal observational data is challenging. Instead of learning the causal structure of an entire dataset, some researchers focus on the analysis of causal relations between two variables only. The state-of-the-art conditional independence-based causal discovery methods (see, e.g., [1,2]) construct graphs that are Markov equivalent, but these methods are not applicable in the case of two variables, since X→Y and Y→X are Markov equivalent.

The statistical and probabilistic causal inference methods based on assumptions of independence of cause and mechanism (see [3] for a general overview) appeared relatively recently and achieve very reasonable empirical results. The main idea behind these methods is as follows: if a simple function that fits data exists, then it is likely that it also describes a causal relation in the data.

The main goal of our paper is to try to reconcile two modern viewpoints on causal inference: the research direction initiated by [1,2], which is based on the assumption of conditional independencies, and the more recent research avenue where the main claim is that causal inference between two observations only is feasible [4,5,6,7,8,9,10], the theory of which relies on the independence of cause and mechanism.

To illustrate the intuition behind our approach, let us consider an example from [3] with altitude and temperature, where *A* is altitude, *T* is temperature, P(A) are city locations, and P(T|A) is the physical mechanism of temperature given altitude, and it can be shown that changing the city locations P(A) does not change the conditional probability P(T|A). The postulate of independence of cause and mechanism allows the causal direction A→T to be inferred. Any latent variables are ignored in this case. However, the city locations depend on a country, since each country has its own urban policy, population density, etc. Thus, in this example, P(A) has at least one latent variable which is county *C*. However, no matter what country is chosen, the physical mechanism P(T|A) holds, and the true underlying causal structure is C→A→T. A country defines the distribution of cities. Having two or more countries leads to a family of distributions. This mixture of probability distributions is independent from P(T|A). Thus, this example also explains what is meant under the independence between probability distributions.

To our knowledge, ref. [11] is the most related recent work to our contribution; however, they consider the case of the pseudo-confounders, where all variables, even confounders, are observed. Our contribution is multi-fold:

- Our main theoretical result is an alternative viewpoint on the recently appeared causal inference algorithms that are based on the independence of cause and mechanism. Here, we follow the simplification used by [3]; however, we are aware that the independence of our interest is between the prior of the cause and the mechanism.
- Our main theoretical results are formulated as Theorems 1 and 2.
- Assuming the existence of the hidden instrumental variables, we propose a novel method of causal inference. Since we consider a bivariate causal inference case where only *X* and *Y* are observed, we also propose an approach to estimate the latent instrumental variables for cases where the cluster assumption for the observed data holds.
- We propose a simple and original method to identify latent confounders.
- We validate our method on a synthetic dataset on which we perform extensive numerical experiments and on the cause-effect benchmark, which is widely used by the causal inference community.

The paper is organized as follows. Section 2 discusses the state-of-the-art methods of bivariate causal inference. Preliminaries on the instrumental variables are provided in Section 3. We consider the role of the instrumental variables for causal inference, and we introduce our approach in Section 4. In Section 5, we discuss the results of our numerical experiments on synthetic and standard challenges. Concluding remarks and perspectives close the paper.

## 2. Related Work

In this section, we discuss the state-of-the-art methods of bivariate causal inference and the corresponding assumptions. In the current work, we focus on a family of causal inference methods which are based on a postulate stating that if X→Y, then the marginal distribution P(X) and the conditional distribution P(Y|X) are independent [8,12,13]. These approaches provide causal directions based on the estimated conditional and marginal probability distributions from observed non-temporal data. One of the oldest and most well-studied types of models describing causal relations that is necessary to mention is structural causal models (SCM). An SCM where X→Y is defined as follows:

(1) $$\begin{array}{cc} X & =N_{X},\;Y=f_{Y}\left(X,N_{Y}\right), \end{array}$$

where $N_{X}$ and $N_{Y}$ are independent. Given $f_{Y}$ and the noise distributions $P_{N_{Y}}$ and $P_{N_{X}}$, we can sample data following an SCM.

A recently proposed but already often used postulate of independence of cause and mechanism is formulated as follows (see, e.g., [8,12,13]). If *X* causes *Y*, then P(X) and P(Y|X) estimated from observational data contain no information about each other. Looking for a parallel between the postulate and the SCM, we assume that in an SCM, $f_{Y}$ and $P_{N_{Y}}$ contain no information about $P_{X}$, and vice versa. The postulate describes the independence of mechanisms and states that a causal direction can be inferred from estimated marginal and conditional probabilities (considered as random variables) from a dataset. In the following, we investigate this research direction.

It is not obvious how to formalise the independence of the marginal and conditional probabilities. A reasonable claim [3] is that an optimal measure of dependence is the algorithmic mutual information that relies on the description length in the sense of Kolmogorov complexity. Since the exact computations are not feasible, there is a need for a practical and reliable approximation. Such an approximation encodes that P(X) and P(Y|X) require more compact models in a causal direction and more complex models in an anticausal direction.

Two families of methods of causal inference dealing with bivariate relations are often discussed. For a more general overview of causal structure learning see [3,14]. Additive noise models (ANM) introduced by [15,16] are an attempt to describe causal relations between two variables. The ANMs assume that if there is a function *f* and some noise *E*, such that Y=f(X)+E, where *E* and *X* are independent, then the direction is inferred to be X→Y. A generalised extension of the ANM, called post-nonlinear models, was introduced by [17]. However, the known drawback of the ANM is that the model is not always suitable for inference on discrete tasks [18].

Another research avenue exploiting the asymmetry between cause and effect is the linear trace (LTr) method [19] and information-geometric causal inference (IGCI) [13]. If the true model is X→Y, and if P(X) is independent from P(Y|X), then the trace condition is fulfilled in the causal direction and violated in the anticausal one. The IGCI method exploits the fact that the density of the cause and the log slope of the function transforming cause to effect are uncorrelated. However, for the opposite direction, the density of the effect and the log slope of the inverse of the function are positively correlated. The trace condition is proved under the assumption that the covariance matrix is drawn from a rotation invariant prior [12]. The method was generalized for non-linear cases [20], and it was shown that the covariance matrix of the mean embedding of the cause in reproducing kernel Hilbert space is free independent with the covariance matrix of the conditional embedding of the effect given cause. The application of the IGCI to high-dimensional variables is considered in [19,21]. Here, the independence between probability distributions is based on the trace condition. The identifiability via the trace condition is proved [3,21] for deterministic relations, and no theory exists for noisy cases, which are much more relevant for real-life applications.

Origo [22] is a causal discovery method based on the Kolmogorov complexity. The minimum description length (MDL) principle can be used to approximate the Kolmogorov complexity for real tasks. Namely, from an algorithmic information viewpoint, if X→Y, then the shortest program that computes *Y* from *X* is more compact than the shortest program computing *X* from *Y*. The obvious weakness of methods based on the Kolmogorov complexity, and also of Origo, is that the MDL only approximates Kolmogorov complexity and involves unknown metric errors that are difficult to control. The empirical performance is highly dependent on a dataset, and Origo was reported to reach state-of-the-art performance on the multivariate benchmarks (acute inflammation, ICDM abstracts, adult dataset); however, it performs less accurately than the ANM on the bivariate benchmark of cause-effect pairs with known ground truth (the Tübingen data set) [23]. We also use this benchmark for our experiments.

There exist various applications of causal inference. Thus, [24] provides a geometric interpretation of information flow as a causal inference. Speaking of probabilistic causal inference approaches, we would like to mention [25], which is a survey considering probabilistic causal dependencies among variables. Information theory is used in [26] to apply bivariate analysis to discover the causal skeleton for multivariate systems. Note that the method which is proposed in our contribution can also be extended to a multivariate case in a similar way.

The most studied causal inference case is probably the case of time series [27], where the Granger causality can be applied. We would like to underline that we consider the case of observational non-temporal data in the current contribution, and the results on the time series are beyond the scope of our paper.

We would like to underline the differences between [11] and our results. The researchers consider a surrogate variable related to a distribution shift that characterises hidden quantities that imply changes across domains and/or time. It is reported that it is possible to find causal models in each domain or for each time point for non-stationary data, but they propose using the information on the distribution shift to identify one causal model across domains and/or time. This surrogate variable can be seen as a confounder; however, it is assumed that the values of these confounders are fixed and always observed (Assumption 1 and Section 3.2 of [11]). Thus, they are pseudo-confounders. We, on the contrary, assume that the surrogate variable is not observed, and we do not assume that the confounders exist. We pose a challenge to identify their existence and to approximate latent instrumental variables.

## 3. Independence of Probability Distributions and Instrumental Variables

Let *X* and *Y* be two correlated variables. In the settings considered by [3], in order to decide whether X→Y or Y→X, it is proposed to check if the distributions P(X) and P(Y|X) are independent. As far as we know, this independence between distributions (and not between random variables) does not have any formal definition. However, some useful properties can be derived, and various criteria were constructed for different cases [4,5,6,7,8,9]. In this paper, we adopt the following definition. Let P(X,Y) be the joint distribution of X,Y in a population P; let Q(X,Y) be the joint distribution of X,Y in another population Q. If *X* is the cause of *Y*, the causal mechanism should be the same in the two distributions:

(2) $$\begin{array}{c} P(X,Y)=P(X)\cdot P(Y|X), \end{array}$$

(3) $$\begin{array}{c} Q(X,Y)=Q(X)\cdot P(Y|X), \end{array}$$

i.e., P(Y|X)=Q(Y|X), and on the contrary, P(X|Y)≠Q(X|Y). More generally, for all mixed populations between P and Q, and for all mixtures $Q_{\lambda }=\lambda P+\left(1-\lambda \right)Q$ with λ∈[0,1]:

(4) $$\begin{array}{c} \forall \lambda \in \left[0,1\right],Q_{\lambda }\left(X\right)⫫Q_{\lambda }\left(Y|X\right) \end{array}$$

(5) $$\begin{array}{c} \Leftrightarrow Q_{\lambda }\left(Y|X\right)=P\left(Y|X\right). \end{array}$$

Now, we consider λ as a hyper-parameter for a (latent) prior $I_{X}$ that allows the population ($P\left(X|I_{X}=0\right)=P\left(X\right)$, $P\left(X|I_{X}=1\right)=Q\left(X\right)$) to be selected. In this meta-model, $I_{X}$ and *X* are dependent, and *X* and *Y* are dependent. However, $I_{X}$ and *Y* are independent conditionally to *X*. On the contrary, if we consider λ as a hyper-parameter for a (latent) prior $I_{Y}$, this allows the population ($P\left(Y|I_{Y}=0\right)=P\left(Y\right)$, $P\left(Y|I_{Y}=1\right)=Q\left(Y\right)$) to be selected. In this meta-model, $I_{Y}$ and *Y* are dependent, and *X* and *Y* are dependent. However, since P(X|Y)≠Q(X|Y), $I_{Y}$ and *X* are not independent, even conditionally to *Y*.

To provide some intuition behind such a mixture model, let P(X) and Q(X) be the distributions of city locations in two different countries and P(Y|X) be a physical mechanism predicting weather in a given location. Then λ is the hyper-parameter controlling the proportion of observations in each country, and note that λ, P(X), and Q(X) are independent from P(Y|X). Such a representation of the problem as a mixture model with latent priors motivates our proposition to use models with instrumental latent variables.

The aim of models with instrumental variables [28,29,30] where *X*, *Y*, and $I_{X}$ are observed, and *U* is an unobserved confounder, is to identify the causal effect of *X* on *Y*. Assuming that the relationships are linear, and applying a linear Gaussian structural causal model, one can write

(6) $$\begin{array}{cc} X & =\alpha_{0}+\alpha I_{X}+\delta U+\epsilon_{X}, \end{array}$$

(7) $$\begin{array}{cc} Y & =\beta_{0}+\beta X+\gamma U+\epsilon_{Y}, \end{array}$$

where $\epsilon_{X}$ and $\epsilon_{Y}$ are noise terms, independent of each other. It is assumed, without loss of generality, that *U*, $\epsilon_{X}$, and $\epsilon_{Y}$ have mean zero. Note that the common cause *U* can be absent, and we are not going to assume that *U* exists when modelling dependencies between *X* and *Y*. The instrumental variable $I_{X}$ is uncorrelated with ancestors of *X* and Y. The instrumental variable is a source of variation for *X*, and it only influences *Y* through *X*. Studying how *X* and *Y* respond to perturbations of $I_{X}$ can help one deduce how *X* influences *Y*. A two-stage least squares [31] can be used to solve the problem.

**Probability distributions as random variables**

Similar to [3,21], we consider probability distributions as random variables. P(X) is a function of X∈[0,1], and thus, they are random variables distributed in [0,1]. Note that a model where a probability is randomly generated is an example of a hierarchical model, or of a model with priors, where some parameters are treated as random variables.

## 4. Latent Instrumental Variables for Causal Discovery

In this section, we show that the methods based on the independence of cause and mechanism, introduced by [4,5,6,7,8,9], indirectly contain traces of the existence of the hidden instrumental variable. This can be seen as follows. P(X) generates *X* in the approaches proposed and investigated by the scientists mentioned above. In our method, we assume that *X* are generated by $I_{X}$. Therefore, there is a strong parallel between P(X) and $I_{X}$, which are both priors for the observations. Thus, our method described below also provides some intuition and interpretation of the recently proposed algorithms based on the independence between the “cause and the mechanism”. We provide some theoretical results on the independence of the causal mechanisms in terms of probability distributions and information theory. These results allow us to derive a novel algorithm of causal inference which is presented in the section below.

Our observations are *X* and *Y*, two one-dimensional vectors of the same length *N*, and these variables are correlated. Here, we suppose that either causality between these variables exists, and either X→Y, Y→X, or a common latent cause X←U→Y can be identified, where *U* is a hidden variable that can impact *X* and/or *Y*. Let $I_{X}$ and $I_{Y}$ denote latent instrumental variables of *X* and *Y*, respectively. In the current contribution, we do not observe the instrumental variables; we assume that they exist and can be approximated. We do not assume that the common cause *U* exists; however, we show how its existence can be deduced, if this is the case.

**Figure 1:**
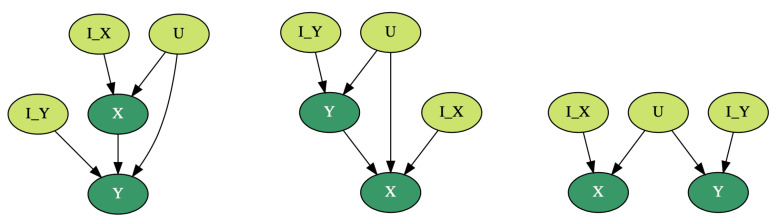
The models of our interest. The dark nodes are observed from data, and the light coloured nodes are latent.

There are three graphical structures that are of particular interest for us. They are shown on Figure 1: the dark nodes are observed, and the instrumental variables and the common latent cause are not observed from data.

**Assumption** **1.** *In the case of observational non-temporal data, if $I_{X}$ exists such that $I_{X}\to X$, and if $I_{Y}$ exists such that $I_{Y}\to Y$, and if the random variables X and Y are correlated, then we assume that it is impossible that both $I_{X}⫫Y|X$ and $I_{Y}⫫X|Y$ hold.*

**Theorem** **1.** *Let X and Y be two correlated random variables, and they do not have any common cause. We assume that either X causes Y, or vice versa. If there exists a random variable $I_{X}$ such that $I_{X}\to X$, and if $I_{X}⫫Y|X$, then we are able to infer causality and decide that X→Y.*

**Proof.**  Several directed acyclic graphs (DAGs) may be Markov equivalent [1,2]. We assume that once an essential graph is found, the directed arcs of this graph are interpreted causally.

Under the assumption that $I_{X}\to X$, and if $I_{X}⫫Y|X$, the only possible directed graph is $I_{X}\to X\to Y$. In the case where IX⫫Y|X, we obtain $I_{X}\to X\leftarrow Y$.    □

**Theorem** **2.** *If the true causal structure is $I_{X}\to X\to Y$, and X and Y do not have any common cause, then P(Y|X) does not contain any information about P(X), and vice versa; however, P(X|Y) and P(Y) are not independent.*

**Proof.**  Assume that $I_{X}⫫Y|X$. Let us consider the relation between P(Y|X) and P(X). In the following, we treat P(Y|X), P(X|Y), P(X), and P(Y) as random variables. We can write

(8) $$\begin{array}{c} P\left(Y|I_{X},X\right)=P\left(Y|X\right). \end{array}$$

Note that we do not have P(X) in Equation (8) when we express P(Y|X) for $I_{X}\to X\to Y$. Let us consider the relation between P(X|Y) and P(Y) for the same graphical structure. We obtain

(9) $$\begin{array}{c} P\left(X|Y\right)=\frac{P\left(Y|X\right)P\left(X|I_{X}\right)}{P(Y)}, \end{array}$$

where the form of the nominator is due to the fixed dependencies $I_{X}⫫Y|X$. From Equation (9), we clearly see that P(X|Y) is not independent from P(Y) for this graphical structure.    □

Table 1 provides the state-of-the-art methods of the bivariate causal inference (left column) and the corresponding equivalent models with the latent instrumental variables $I_{Y}$ and $I_{X}$, if they can be reconstructed (right column).

**Construction of the Instrumental Variables**

**Assumption** **2.** *(Cluster assumption. [33]) If points are in the same cluster, they are likely to be in the same class.*

In some tasks, the instrumental variables (IV) are observed, and their application is straightforward. In a number of applications, they are not provided. Here, we discuss how the instrumental variables can be approximated, and we draft a procedure to estimate them. In our experiments, in Section 5, we apply the proposed method for the IV construction. Note that the identification and characterisation of latent variables is a challenge in itself. Our work is slightly similar to [34,35] in that we apply clustering methods to create the latent variables. Taking into account that only *X* and *Y* are observed, the instrumental variables can be constructed using either *X*, *Y*, or both, and an optimal choice of the variables (X, Y, or both) that are related to the IV is in its turn related to a graphical structure that we try to identify and to orient. Thus, for a structure $I_{X}\to X\to Y$, $I_{X}$ does not contain information about *Y*, and $I_{X}$ has to be constructed from *X* only. On the contrary, in the case of $X\to Y\leftarrow I_{Y}$, $I_{Y}$ is not independent from *X*, and $I_{Y}$ has to contain information about both *X* and *Y*.

We rely on clustering methods for the instrumental variables estimation. In our experiments, we apply the k-means clustering; however, other clustering approaches can be used. Algorithm 1 drafts the procedure to approximate the candidates for the IV. We developed a method—Algorithm 2—that makes the decision of whether $I_{X}$ and $I_{Y}$ are to be constructed from one or two observed variables. The proposed algorithm constructs the instrumental variables separately from *X*, *Y* ( $I_{X_{X}}$, $I_{Y_{Y}}$), and from both ($I_{X_{XY}}$, $I_{Y_{YX}}$), and tests which instrumental variables are more relevant. Algorithm 2 compares the distance (we considered the Euclidean distance in our experiments; however, another measure, e.g., the Kullback–Leibler, can be used) between $I_{X_{X}}$ and $I_{X_{XY}}$, and between $I_{Y_{Y}}$ and $I_{Y_{YX}}$. The intuition behind the proposed criterion is as follows. If *Y* influences clustering of *X* less than *X* impacts clustering of *Y* (the condition $if(\mathrm{dist}\left(I_{X_{X}},I_{X_{X}Y}\right)<\mathrm{dist}\left(I_{Y_{Y}},I_{Y_{Y}X}\right))$ in Algorithm 2), then we apply $I_{X}$ constructed from *X* only, and $I_{Y}$ is constructed from *X* and *Y*. Furthermore, vice versa. An important remark is that this criterion has a lot in common with the causal discovery methods based on the Kolmogorov complexity and the MDL: to infer causality, our criterion choses a simpler model.

**A Symmetric Causal Inference Algorithm**

**Figure 2:**
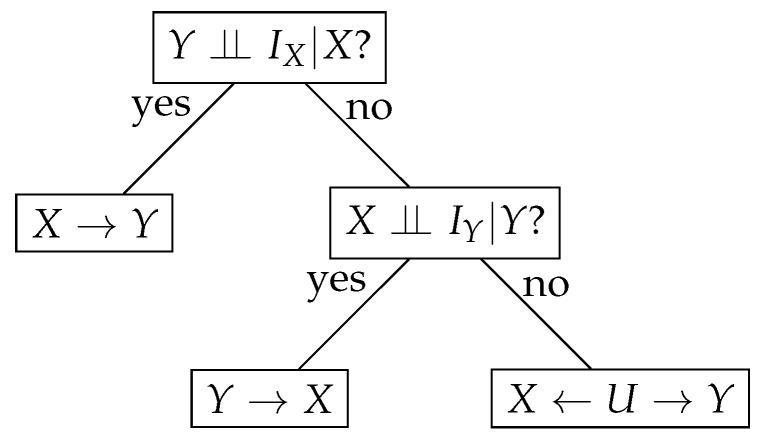
A symmetric causal inference algorithm.

We introduce a simple symmetric algorithm based on the conditional (in)dependence tests to infer causality. It relies on the theoretical foundations provided above. Our algorithm is sketched as a decision tree in Figure 2. It takes $I_{X}$, $I_{Y}$, *X*, and *Y* and returns a causal direction. Precisely, if a conditional independence test states that $Y⫫I_{X}|X$ is true, then X→Y is inferred; otherwise, we test whether $X⫫I_{Y}|Y$, and if it is true, then *Y* causes *X*. The last case where *X* and *Y* are correlated but both $Y⫫I_{X}|X$ and $X⫫I_{Y}|Y$ are false, let us conclude that there is a common hidden cause *U*, and Y←U→X.
**Algorithm 1** Construction of IV Candidates$I_{X_{X}}$ (IV variable of *X* from *X*)
Fix a number of clusters *K*
Cluster ${\left\{X_{i}\right\}}_{i=1}^{N}$ into *K* clusters
**for**
i=1:N
**do**
  $I_{i,X_{X}}$ is the centre of the cluster where $X_{i}$ belongs
**end for**
$I_{X_{XY}}$ (IV variable of *X* from *X* and *Y*)
Fix a number of clusters *K*
Cluster ${\left\{X_{i},Y_{i}\right\}}_{i=1}^{N}$ into *K* clusters
**for**
i=1:N
**do**
  $I_{i,X_{XY}}$ is the 1st coordinate (corresponding to *X*) of the clusters centres where $\left(X_{i},Y_{i}\right)$ belongs
**end for**
$I_{Y_{Y}}$ (IV variable of *Y* from *Y*)
is constructed similarly to the IV variable of *X* from *X*
$I_{Y_{YX}}$ (IV variable of *Y* from *X* and *Y*)
is constructed similarly to the IV variable of *X* from (X,Y)
(Take the 2nd coordinate of the clusters centres)


**Algorithm 2** Approximation of the Instrumental Variables (IV) $I_{X}$ and $I_{Y}$ from *X* and *Y*.**Input:** Observations *X* and *Y*, a clustering algorithm
**Output:** Instrumental variables $I_{X}$ and $I_{Y}$
// Construct instrumental variables to be tested
Construct IV of *X*, $I_{X_{X}}$ using *X* only
Construct IV of *X*, $I_{X_{XY}}$ using *X* and *Y*
Construct IV of *Y*, $I_{Y_{Y}}$ using *Y* only
Construct IV of *Y*, $I_{Y_{YX}}$ using *X* and *Y*
// Take the decision which IV to use
**if**
$\left(\mathrm{dist}\left(I_{X_{X}},I_{X_{XY}}\right)<\mathrm{dist}\left(I_{Y_{Y}},I_{Y_{YX}}\right)\right)$
**then**
  // the IV of *X* is constructed from *X* only
  $I_{X}=I_{X_{X}}$
  // the IV of *Y* is constructed from both *X* and *Y*
  $I_{Y}=I_{Y_{YX}}$
**else**
  // the IV of *Y* is constructed from *Y*
  $I_{Y}=I_{Y_{Y}}$
  // the IV of *X* is constructed from *X* and *Y*
  $I_{X}=I_{X_{XY}}$
**end if**


## 5. Experiments

In this section, we illustrate the predictive efficiency of the proposed method on both artificial and real datasets. We run the numerical experiments on a recent MacBook Pro, 2.6GHz 6-core Intel Core i7, 16GB memory. We use the R language and environment for our experiments, in particular the bnlearn R package.

**Simulated Data**

We consider simple discrete and continuous scenarios. In the discrete case, we fix the structures and the probability distributions on the graphs and generate binary variables. In the continuous case, we use a Gaussian distribution. We generate the instrumental variables $I_{X}$ and $I_{Y}$, *X* and *Y*, and the hidden variable *U*. We use the bnlearn R package to construct the synthetic datasets, and we also use the conditional independence tests from the same package. For our discrete setting with binary variables, we apply an asymptotic mutual information independence test ci.test(test=’mi’), and for the continuous setting with Gaussian variables, we apply the exact *t*-test for Pearson’s correlation ci.test(test=’cor’). Note that the abovementioned conditional independence tests from the bnlearn R package return “big” *p*-values if the variables are conditionally independent, and the *p*-values are small (with an arbitrary threshold 0.05) for dependent variables.

**Figure 3:**
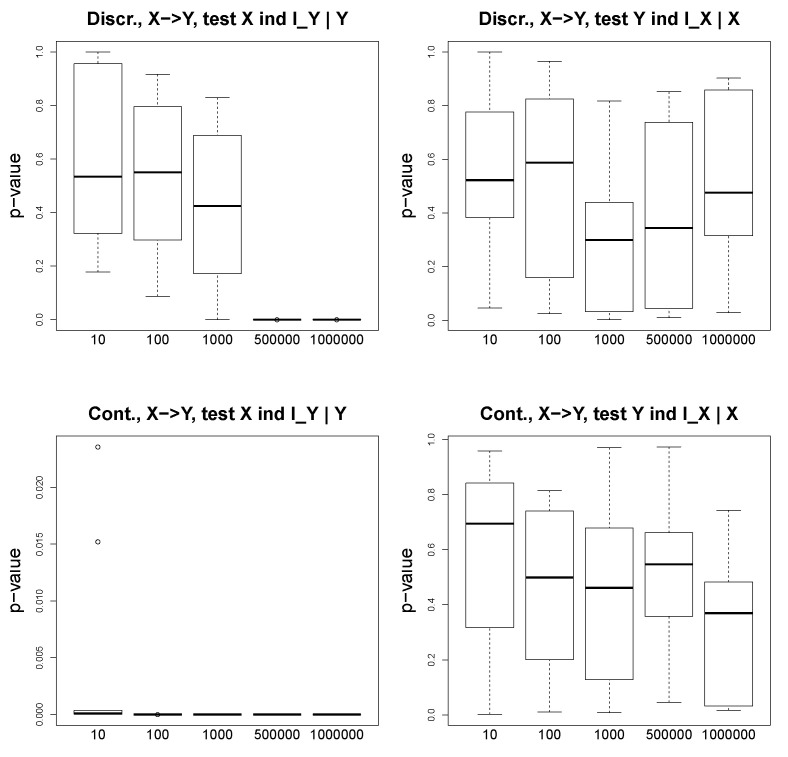
Simulated data. Ground truth: X→Y. Two plots above: discrete data; two plots below: continuous data. The *p*-values of an asymptotic mutual information test (for the discrete case) and an exact *t*-test for Pearson’s correlation (the continuous case) as a function of the number of observations (x-axis).

We consider and simulate discrete and continuous data for two following scenarios: (1) X→Y, and (2) X←U→Y. We test a various number of observations, from 10 to 10,000, and we observe that in the discrete case, even for such a simple problem as one with variables taking binary values, a large number of observations is needed to obtain a reasonable performance. Figure 3 illustrates the *p*-values of the conditional independence tests for the discrete (two plots above) and continuous (two plots below) settings. We show the results for both cases $X⫫I_{Y}|Y$ and $Y⫫I_{X}|X$. We observe that for the ground truth X→Y, $X⫫I_{Y}|Y$ asymptotically converges to small *p*-values (close to 0), and $Y⫫I_{X}|X$ returns large *p*-values, even for a large number of observations.

**Figure 4:**
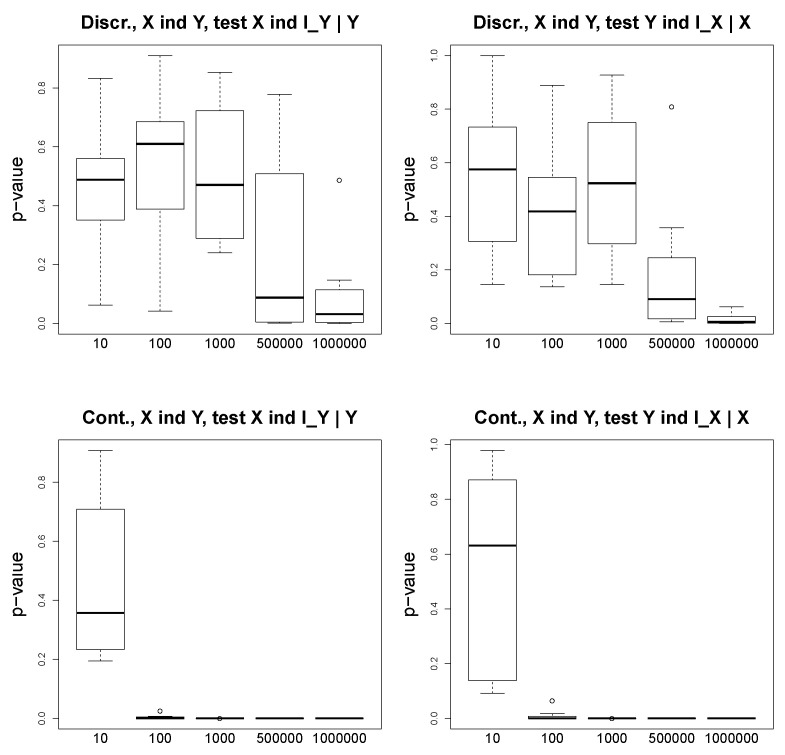
Simulated data. Ground truth: X⫫Y|U. Above: two plots for the discrete setting; below: two plots for the continuous setting. The *p*-values as a function of the number of observations (x-axis).

**Figure 5:**
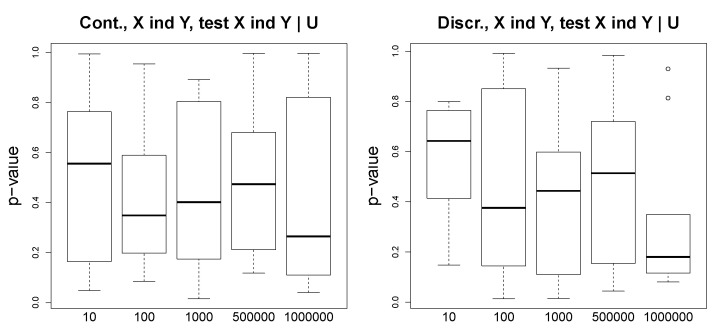
Simulated data. Ground truth: X⫫Y|U. The results of the conditional independence tests for X⫫Y|U for continuous (on the left) and discrete (on the right) data. On the x-axis: the number of observations.

Figure 4 shows our results for the scenario X←U→Y. For the discrete and continuous experiments, we test whether $Y⫫I_{X}|X$ and whether $X⫫I_{Y}|Y$. We see that the variables are not independent. In Figure 5, we demonstrate the *p*-values of the conditional independence test Y⫫X|U, which is a sanity check, and we observe that in this case where the ground truth is X←U→Y, the *p*-values are far from 0 for both continuous and discrete scenarios. In the experiments on the simulated data, our aim is to show that the *p*-values are reasonable indicators of the conditional independence. We do not report the accuracy values, since it is straightforward according to the proposed algorithm (Figure 2).

**Cause-Effect Pairs**

**Figure 6:**
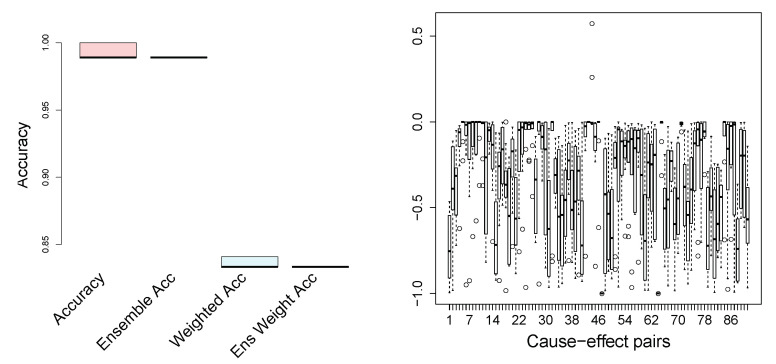
On the **left**: accuracy on the cause-effect benchmark. On the **right**: the difference between the test statistics $X⫫I_{Y}|Y$ and $Y⫫I_{X}|X$.

We tested the proposed algorithm on the benchmark collection of the cause-effect pairs, obtained from http://webdav.tuebingen.mpg.de/cause-effect (accessed on 15 January 2021), version 1.0. The data set contains 100 pairs from different domains, and the ground truth is provided. The goal is to infer which variable is the cause and which is the effect. The pairs 52–55, 70–71, and 81–83 are excluded from the analysis, since they are multivariate problems. Note that each pair has an associated weight, provided with the data set, since several cause-effect pairs can come from the same scientific problem. In a number of publications reporting results on this dataset, the accuracy is a weighted average. We apply the proposed method, described in Section 4, to infer causality on the cause-effect pairs. In Figure 6, we show the standard (unweighted) accuracy and the weighted accuracy, where the weights for each observation pair are given in the dataset. To increase the stability and also the accuracy, we propose a scenario where we split the data into k-folds, carry out causal inference on each fold separately, and take an ensemble decision on the causal direction. The accuracy for such an ensemble approach is also shown in Figure 6 for both weighted and not weighted performance. The number of folds in our experiments is 10. Speaking of state-of-the-art results on the cause-effect pairs, it was reported that Origo [22] achieves (weighted) accuracy of 58%, and the ANM [16] reaches 72±6%. Figure 6 illustrates that the proposed method outperforms the state-of-the-art algorithms: our weighted accuracy is 83.2%. Note that the ensemble method reduces the variance significantly. We do not provide the results of the extensive numerical comparisons of the state-of-the-art methods on the cause-effect pairs, since these results can be easily found in the original papers (cited in the Related Work section). Moreover, the goal of the current work is not only to achieve state-of-the-art results and to outperform them, which we do, but also to focus on an alternative formulation of the independence of the cause and the causal mechanism, as well as to consider a reasonable method for the identification and construction of the hidden instrumental variables.

What is central and what is interesting to look at are the *p*-values of the conditional independence tests (here, the exact *t*-test for Pearson’s correlation from bnlearn R package) $X⫫I_{Y}|Y$ and $Y⫫I_{X}|X$. In Figure 6 (on the right), we show their difference. If the *p*-values of the test $X⫫I_{Y}|Y$ are small (that is, *X* and $I_{Y}$ are not independent, given *Y*) and the results of $Y⫫I_{X}|X$ are relatively large (or larger than ones of $X⫫I_{Y}|Y$), stating that *Y* and $I_{X}$ are independent, given *X*, then the plotted difference is negative. This is exactly what is observed for almost all cause-effect pairs.

Figure 6 (on the right) shows our results for the case where the number of clusters, i.e., modalities of the hidden instrumental variables, is set to 15 for both $I_{X}$ and $I_{Y}$. We tested different numbers, *K*, of clusters for the construction of instrumental variables (see Section 4 for details). For the current task, we did not notice any important impact on the result; however, taking extremely small (2–3) and large (70–100) numbers of clusters degrades the performance. In practical real applications, an optimal *K* can be fixed using a grid search.

## 6. Conclusions, Limitations, and Future Research

We posed a challenge to bring together two principle research avenues in causal inference: causal inference using conditional independence and methods based on the postulate of independence of cause and mechanism. We focused on the methods of causal inference based on the independence of cause and mechanism, and we provided some theoretical foundations for this family of algorithms. Our main message is that the role of the hidden instrumental variables cannot be neglected.

The implications of our study are twofold. First, the proposed method will motivate the development of novel theoretical (probabilistic) approaches to recover hidden common causes. Second, our method can already be tested and studied for some real biological and medical applications. However, the application to real problems, especially to medical and biological tasks, should be done in tight collaboration with human experts.

We propose an algorithm to estimate the latent instrumental variables efficiently. We also introduce a simple (and symmetric) algorithm to perform causal inference for the case of two observed variables only, where the corresponding instrumental variables are approximated. Our original approach is simple to implement, since it is based on a clustering algorithm (we used the k-means; however, any other clustering method can be tested) and on conditional independence tests. The introduced approach can be applied to both discrete and continuous data, and we have shown that it is extremely competitive compared to the state-of-the-art methods on a real benchmark, where a cluster assumption holds.

The main limitation of our work is that it is focused on the bivariate case; however, in a number of real applications, there is a need to infer causality between several variables.

Currently, we consider an extension of the proposed algorithm to more complex graphs and potentially huge applications, such as modelling gene interactions. Another avenue of research is novel metrics to measure the conditional independence of variables.

## Figures and Tables

**Table 1:** Some state-of-the-art methods for causal discovery for the ground truth X→Y, under the assumption that IX⫫Y|X, and the corresponding models with the latent instrumental variables.

The state-of-the-art methods of bivariate causal inference and their main ideas	Existence of hidden instrumental variables, an equivalent model with the latent IV
CURE (unsupervised inverse regression) [8]:	This implies directly that $P(X|I_{Y},Y)$, X⫫IY|Y,
It is possible to recover P(X|Y) from P(Y),	and therefore, $I_{Y}$ is needed to recover
it is not possible to recover P(Y|X) from P(X)	the conditional probability
Information-geometric approach [13]:	$\mathrm{cov}\left(P\left(Y|I_{X},X\right),P\left(X\right)\right)=\;0$
$\mathrm{cov}\left(\log f^{\prime },P\left(X\right)\right)\;=\;0,\mathrm{cov}\left(\log f^{-1^{\prime }},P\left(Y\right)\right)\;\geq \;0,$	$\mathrm{cov}\left(P\left(X|I_{Y},Y\right),P\left(Y\right)\right)\geq \;0$
$f^{\prime }$ is log slope of the func. transform.	$\mathrm{cov}\left(P(Y|X),P(X)\right)=0$
cause to effect	$\mathrm{cov}\left(P\left(X|I_{Y},Y\right),P\left(Y\right)\right)\geq 0$
Comparing regression errors [32]:	$E\left[\mathrm{var}\left(Y|I_{X},X\right)\right]\leq E\left[\mathrm{var}\left(X|I_{Y},Y\right)\right]$
$E\left[{\left(Y-E\left[Y|X\right]\right)}^{2}\right]\leq E\left[{\left(X-E\left[X|Y\right]\right)}^{2}\right]$	$E\left[\mathrm{var}\left(Y|X\right)\right]\leq E\left[\mathrm{var}\left(X|I_{Y},Y\right)\right]$
Using the distance correlation [9]:	
$D\left(P\left(X\right),P\left(Y|X\right)\right)\leq D\left(P(Y),P(X|Y)\right)$,	$D\left(P\left(X\right),P\left(Y|I_{X},X\right)\right)\leq D\left(P\left(Y\right),P\left(X|I_{Y},Y\right)\right)$
where D is distance correlation	$D\left(P(X),P(Y|X)\right)\leq D\left(P\left(Y\right),P\left(X|I_{Y},Y\right)\right)$
Via kernel deviance measures [10]: $S_{X\to Y}=$	Compare (μ is cond. mean embedding)
$\frac{1}{N}\sum_{i=1}^{N}{\left(∥\mu_{Y|X=x_{i}}{∥}_{H_{y}}-\frac{1}{N}\sum_{j=1}^{N}{∥\mu_{Y|X=x_{j}}∥}_{H_{y}}\right)}^{2}$	$\frac{1}{N}\sum_{i=1}^{N}{\left(∥\mu_{Y|I_{X},X=x_{i}}{∥}_{H_{y}}-\frac{1}{N}\sum_{j=1}^{N}{∥\mu_{Y|I_{X},X=x_{j}}∥}_{H_{y}}\right)}^{2}$ vs.
$H_{y}$ – RKHS, $S_{Y\to X}$ analogously, $S_{X\to Y}\leq S_{Y\to X}$	$\frac{1}{N}\sum_{i=1}^{N}{\left(∥\mu_{X|I_{Y},Y=y_{i}}{∥}_{H_{x}}-\frac{1}{N}\sum_{j=1}^{N}{∥\mu_{X|I_{Y},Y=y_{j}}∥}_{H_{x}}\right)}^{2}$

## Data Availability

The data used in the experiments are publicly available, and can be downloaded from http://webdav.tuebingen.mpg.de/cause-effect.
